# Theories to Explain Exercise Motivation and Physical Inactivity: Ways of Expanding Our Current Theoretical Perspective

**DOI:** 10.3389/fpsyg.2019.01147

**Published:** 2019-05-21

**Authors:** Ralf Brand, Boris Cheval

**Affiliations:** ^1^Sport and Exercise Psychology, University of Potsdam, Potsdam, Germany; ^2^Swiss Center for Affective Sciences, University of Geneva, Geneva, Switzerland; ^3^Laboratory for the Study of Emotion Elicitation and Expression (E3Lab), Department of Psychology, University of Geneva, Geneva, Switzerland

**Keywords:** exercise, motivation, affect, automaticity, physical inactivity

Physical inactivity and lack of exercise are major societal health problems. Most experts in exercise psychology, if asked how to support people in growing their motivation for physical activity and exercise, would probably recommend shifting the decisional balance by creating a belief that there are more benefits to be had from becoming active than barriers to be overcome, bolstering their appraisals of self-efficacy, and creating social environments that promote perceptions of autonomy, competence, and relatedness (e.g., Biddle and Vergeer, 2019). These recommendations are evidence-based (e.g., Teixeira et al., 2012; Young et al., 2014). Many empirical studies show that people who are sufficiently physically active differ in these variables from those who are less active. There are also longitudinal and intervention studies demonstrating that changing these motivational variables makes behavior change more likely.

On the other hand, there is growing skepticism as to whether epidemic physical inactivity can be effectively addressed through interventions developed on the basis of the few cognitive-behavioral theories (e.g., Conn et al., 2011) that have been the mainstays in exercise psychology for decades (Rhodes et al., 2019). For example, in the context of a recent congress symposium organized to debate this issue, two renowned researchers explained their conviction and challenged each other with good arguments: One position—that interventions based on these theories provide promising approaches in some contexts, but have proven to be ineffective by and large (Weed, 2018)—contrasted sharply with the other—that results achieved from interventions based on these cognitive-behavioral theories could have been much stronger if only the available evidence had been put into practice more consistently (Hagger, 2018). Other authors have argued that the current situation is forcing a critical reevaluation of cognitive-behavioral theories, since all of them are based on the common assumption that behavioral decisions are driven mainly by the rational evaluation of information (and dramatically neglect the importance of affective and automatic processes; e.g., Ekkekakis, 2017).

We share the concerns that have been expressed. In this text, we will outline our view, focusing on one common element of the most widely used cognitive-behavioral theories of motivation. We will argue that this one element obscures alternative approaches that could lead to more physical activity and exercise in everyday life.

## The Unfavorable Commonality of Cognitive Theories of Physical Activity and Exercise

A framework for classifying theories of exercise and physical activity behavior suggests organizing them into five classes (Biddle et al., 2007). *Belief-attitude theories* focus on the cognitive antecedents of behavioral intentions, defined as the effort someone is prepared to invest toward performing a target behavior. Well-known examples are the theory of reasoned action (Fishbein and Ajzen, 1975) and the theory of planned behavior (Ajzen, 1985). *Competence-based theories* are exemplified primarily by Bandura's construct of self-efficacy, which is defined as an individual's judgment of his or her “capabilities to organize and execute courses of action required to attain designated types of performances” (Bandura, 1986, p. 391). *Control-based theories* refer to the notion that humans have the intrinsic desire or goal to experience oneself as the initiator and regulator of one's actions. The currently highly influential self-determination theory attributes this desire to a basic psychological need for autonomy (Deci and Ryan, 1985). S*tage models*, e.g., the transtheoretical model (Prochaska and DiClemente, 1983), conceptualize behavioral change as a process that brings one closer to the envisaged goal. *Hybrid models*, such as the health action process approach (Schwarzer, 1992), combine the stage concept with motivational variables, to predict intention, with the addition of post-decisional variables (e.g., implementation intention;Gollwitzer, 1999).

All these models have one core attribute in common, stemming from origins of cognitive theorizing in psychology: They all emphasize the importance of imagined end states (behaviors or goals) and the energization of action resulting from them to such an extent that the experience of situated factors (e.g., momentary affect linked to the situation; Ekkekakis, 2017) is overlooked.

This notion that a better understanding of ongoing behavior requires both situated factors and the cognitive projections that direct behavior was proposed by Lewin (1951). He conceptualized every specific state of behavior as either the result of equal but opposite forces, which hold the person in his or her current state, or under the influence of relatively stronger driving forces, which direct the person away from the current state.

This theoretical idea had limited impact on research on motivation however. Probably because the cognitive revolution in psychology, which began soon after Lewin's early death (in 1947) fascinated so many psychologists. With the advent of the contention that people are able to engage in forethought, the separate conceptualization of momentary restraining forces was lost.

Two recent theories in exercise psychology tie in with considerations about situated restraining forces, albeit in different ways. We will briefly describe the relevant aspects of these theories and outline ideas on how future research could yield alternative intervention approaches for public health.

## Affective-Reflective Theory of Physical Inactivity and Exercise

Affective-Reflective Theory (ART) of physical inactivity and exercise (Brand and Ekkekakis, 2018) is a dual-process theory, which assumes that stimuli (e.g., a friend's reminder that you intended to go for a run, or remembering that you had planned to go for a run) trigger automatic associations and a resulting automatic affective valuation of exercise (type-1 process). An automatic affective valuation is the unattended assignment of positive (association with pleasure) or negative (association with displeasure) value to a stimulus, either as the result of repeated exercise-related emotional experiences mediated by cognitive appraisals (e.g., pride, embarrassment) or as a result of repeated experiences of core affective reactions to stimuli (e.g., sense of physical reinvigoration, bodily discomfort). The automatic affective valuation serves as the basis for a controlled, reflective evaluation (type-2 process), which can follow if self-control resources are available. The reflective evaluation draws on propositions about exercise and physical inactivity, derived from previous experience and mental simulation (e.g., anticipation of the affective consequence of actions). Higher-level cognitive operations, such as deliberative reasoning about one's needs and values (Deci and Ryan, 1985) may also contribute to this process. The automatic affective valuation is connected to an action impulse (approach or avoidance), whereas the controlled response can result in action plans.

The ART aims to explain and predict behavior in situations in which people either remain in a state of physical inactivity or initiate action. It assumes that experience, feelings, and thoughts connected with exercise influence whether someone would be willing to undergo physical strain similar to that previously experienced during exercise. Related to the topic of this opinion article, the ART posits that, in the face of an exercise-related stimulus, one's negative affective valuation of exercise will act as a *restraining force* that may counteract any positive cognitive motivational drives toward action (or, on the other hand, if the affective valuation is positive, it will present a driving force and thus make it more likely that the person will change his or her current state of physical inactivity).

## Theory of Energetic Cost Minimization

The theory of energetic cost minimization (TECM; Cheval et al., 2018a,b) assumes that biomechanically efficient behaviors have a rewarding value. It refers to evidence on the multiple neuro-behavioral adaptations that have contributed to the minimization of metabolic costs in the course of human action and during movement (Srinivasan and Ruina, 2006). For example, individuals automatically adapt their step frequency in real time to optimize energy costs (Selinger et al., 2015) and learn to minimize the physical effort required to obtain specific rewards (Skvortsova et al., 2014). This automatic behavioral tendency of effort optimization is theorized to be a neurobiologically anchored process.

The TECM assumes that situational factors (such as one's internal physiological state or external physical environment) may either incentivize the behavioral opportunities to minimize or lead the individual to effectively temper the tendency to reduce energetic cost (Cheval et al., 2019). The availability of cognitive resources can weaken the automatic tendency toward effort optimization (Cheval et al., 2018c, 2019). In sum, the theory conceptualizes the evolutionary inclination to avoid unnecessary physical exertion as a *restraining force* that may hinder the ability of individuals to effectively implement their conscious intention to be physically active.

## Major Similarities and Differences Between the Two Theories

The ART is a psychological theory that relates what we know about people's acute affective responses to exercise (Ekkekakis et al., 2013) and how such experiences can influence the odds of future exercise (Rhodes et al., 2009; Rhodes and Kates, 2015). Simply put, many people (especially untrained individuals and e.g., overweight people) experience negative affect during exercise and this may have a significant negative effect on further exercise engagement. Habitual physical inactivity and exercise avoidance are explained by the ART as learned reactions; they originate from an automatic negative affective valuation of exercise, constituting an important restraining force. Interventions should, therefore, focus on minimizing unpleasant experiences while exercising, and/or should facilitate consistently pleasant experiences during exercise, so that positive automatic affective valuations of exercise can develop.

The TECM has its roots in evolutionary behavioral biology and posits an ever-present tendency (a restraining force) in human behavior toward efficiency in anticipation of potentially exhausting physical activity as well as during physical performance. In the light of this theory, with regard to possible intervention approaches, people should be aware that this tendency exists. Most generally, executive cognitive functioning, e.g., the capacity for self-control should be strengthened. In addition, psychological training (Sheeran et al., 2013) through (for example) evaluative conditioning, attentional bias modification, or approach-avoidance training could be useful, in order to change individuals' automatic reactions to physical activity-related stimuli and reduce the restraining force.

## Conclusion

In our opinion, the fact that most research in exercise psychology pertaining to how people can be motivated to be more physically active is considered through the prism of a few paradigmatically similar cognitivist theories, is problematic. By focusing on the action energizing properties of expectations and goals, the importance of situated processes that can hold the individual back in that particular moment, has been overlooked. However, in our view, such processes represent crucial components of theoretical explanations of both physical activity (and exercise) and physical inactivity. The two highlighted theoretical approaches, the ART and the TECM, are examples that illustrate directions toward which the field of exercise psychology can evolve.

## Hands-on Recommendations for Real-Life Interventions

Since both theories are relatively new, high-quality studies that would provide direct evidence for the effectiveness of derived intervention methods do not yet exist. Nevertheless, we take the liberty of providing a few suggestions for practice, because these will certainly not have a negative effect on the effectiveness of standard intervention methods (Howlett et al., 2019).

With regard to the ART, we like to emphasize that behavioral interventions (i.e., those that are not only conversation-based) should be delivered by well-trained instructors with expertise in tailored exercise load control. Convincing exercise novices with the help of supportive verbal communication may be a field for psychologists. Facilitating experiences through exercise is profession of exercise specialists however.

With regard to the TECM, it is assumed that the behavioral tendency to minimize can be most effectively counteracted by strengthening the individual's cognitive resources and self-control capacity. Feeling relaxed, energetic and focused should help to more effectively implement plans and intentions. Creating conditions that maximize pleasure during physical activity and exercise may play an additional role in counteracting the tendency to reduce energetic cost.

Generally speaking, we also believe that public policy should endorse open, safe and well-maintained infrastructure to promote access to places for walking, cycling and other physical activities, and the architecture of buildings should encourage physical activity throughout the day (e.g., access to stairs, active work-stations). A multi-faceted approach is warranted to effectively address the pandemic of physical inactivity in everyday life.

## Author Contributions

All authors listed have made a substantial, direct and intellectual contribution to the work, and approved it for publication.

### Conflict of Interest Statement

The authors declare that the research was conducted in the absence of any commercial or financial relationships that could be construed as a potential conflict of interest.
